# Supplementary material for: Attitudes of nursing degree students towards end of life processes. A cultural approach (Spain-Senegal)
Source: PLoS One. 2021 Aug 20;16(8):e0254870. doi: 10.1371/journal.pone.0254870 (PMC8378746; doi:10.1371/journal.pone.0254870)
Supplement: Attachment — Submitted filename: Attitudes towards end of life-review.docx [file pone.0254870.s002.docx]

Introduction

I enjoyed reading the introduction and I congratulate the authors for delivering it in such a nice way. However, I have few comments:

- There is no clue in the abstract that the authors are examining nursing students’ attitudes towards death and end of life during COVID-19. I think highlighting this in the abstract is important.
- It would be beneficial to talk more about nursing students’ preparation towards end-of-life aspects in the University of Huelva (Spain). What the end of life curriculum at the university entails?
- I would suggest to clarify how the Spanish culture compared to that of Senegal view death and what are the cultural attitudes towards death in both?

Methods

- The authors mentioned that the design is observational, and this entails performing some form of observation to students although there is no indication that students’ observation was performed!! Please revise the design of the study to reflect the actual participants’ selection and data collection
- What is meant by multicenter design? Selecting nursing students to participate in the study does not apply to the definition of multicenter design
- The authors mentioned that “Data collection was performed using systematic random sampling, stratified according to university and academic year.” At the same time in the description of the sample, it is obvious that the sample was convenient which is different than systematic random sampling , please revise.
- How the researchers obtained the names of students who regularly attended classes?
- more clear and specific description of the instrument dimensions is needed and to give an item example of each dimension.

Results

- What NR in Table 1 stands for?
- Need to cite the tables in text

Discussion

- Well-written and enjoyed reading it.
- Any recommendations for future research?

-I would suggest to have a section that discuss the limitations of the study.

Overall, Nice work!!
